# Simulating Intestinal Growth Conditions Enhances Toxin Production of Enteropathogenic *Bacillus cereus*

**DOI:** 10.3389/fmicb.2017.00627

**Published:** 2017-04-12

**Authors:** Nadja Jeßberger, Corinna Rademacher, Viktoria M. Krey, Richard Dietrich, Ann-Katrin Mohr, Maria-Elisabeth Böhm, Siegfried Scherer, Monika Ehling-Schulz, Erwin Märtlbauer

**Affiliations:** ^1^Department of Veterinary Sciences, Faculty of Veterinary Medicine, Ludwig-Maximilians-Universität MünchenOberschleißheim, Germany; ^2^Functional Microbiology, Department of Pathobiology, Institute of Microbiology, University of Veterinary Medicine ViennaVienna, Austria; ^3^Lehrstuhl für Mikrobielle Ökologie, Zentralinstitut für Ernährungs- und Lebensmittelforschung, Wissenschaftszentrum Weihenstephan, Technische Universität MünchenFreising, Germany

**Keywords:** *Bacillus cereus*, enterotoxins, simulated intestinal conditions, toxic potential, CaCo-2 cells

## Abstract

*Bacillus cereus* is a ubiquitous bacterial pathogen increasingly reported to be the causative agent of foodborne infections and intoxications. Since the enterotoxins linked to the diarrheal form of food poising are foremost produced in the human intestine, the toxic potential of enteropathogenic *B. cereus* strains is difficult to predict from studies carried out under routine cultivation procedures. In this study, toxigenic properties of a panel of strains (*n* = 19) of diverse origin were compared using cell culture medium pre-incubated with CaCo-2 cells to mimic intestinal growth conditions. Shortly after contact of the bacteria with the simulated host environment, enterotoxin gene expression was activated and total protein secretion of all strains was accelerated. Although the signal stimulating enterotoxin production still needs to be elucidated, it could be shown that it originated from the CaCo-2 cells. Overall, our study demonstrates that the currently used methods in *B. cereus* diagnostics, based on standard culture medium, are not allowing a conclusive prediction of the potential health risk related to a certain strain. Thus, these methods should be complemented by cultivation procedures that are simulating intestinal host conditions.

## Introduction

Due to the secretion of various toxins linked to gastrointestinal as well as non-gastrointestinal diseases, *Bacillus cereus* presents a serious public health hazard (Stenfors Arnesen et al., 2008; Bottone, 2010). Since it is ubiquitous in nature and due to its capability to form stress resistant spores, it is not totally avoidable in certain food production and processing chains. Two different types of food poisoning are known, the diarrheal and the emetic syndrome. The emetic type, which manifests in nausea and vomiting, is caused by the heat-stable cyclic dodecadepsipeptide cereulide, which is produced in foods even before consumption (Agata et al., 1995; Ehling-Schulz et al., 2004; Messelhäusser et al., 2014). The onset of symptoms linked to the emetic toxin cereulide occurs rapidly within 0.5–6 h while the diarrheal type has a long incubation time lasting 5–16 h (Ehling-Schulz and Messelhäusser, 2012). Cause of the latter type of illness, which is associated with a wide range of foods such as milk and milk products, salad, or meat, are enteropathogenic strains that produce heat-labile enterotoxins after outgrowth of the ingested *B. cereus* spores in the human intestine (Clavel et al., 2004). Most important are the two three-component toxin complexes hemolysin BL (Hbl) (Beecher et al., 1995) and the non-hemolytic enterotoxin Nhe (Lund and Granum, 1996; Fagerlund et al., 2010; Didier et al., 2012, 2016). The third known enterotoxin is the single protein CytotoxinK (Cyt K) (Lund et al., 2000). So far, the gene encoding the highly toxic variant CytK1 has been identified in very few strains, which were suggested as the separate species *Bacillus cytotoxicus* (Guinebretière et al., 2013).

Enterotoxin gene expression in *B. cereus* is a highly complex and multifactorial process integrating a vast number of environmental, nutritional, and intrinsic signals leading to an elaborate adaptation of gene transcription to the metabolic status of the cell. The global virulence regulator PlcR activates transcription of the enterotoxin genes during transition state in response to increasing cell density (Gohar et al., 2008). Maximal toxicity during the exponential growth phase has also been observed (Duport et al., 2004; Zigha et al., 2006). The PlcR regulon further comprises genes encoding cereolysin O, hemolysin III, as well as the three phospholipases C (PC-PLC, PI-PLC, SMase) and the immune inhibitor A2 protease (InhA2) (Gohar et al., 2002). *B. cereus* virulence is further closely associated with flagella and motility, aeration, the oxidation–reduction potential (ORP), and nutrients such as the carbon source or iron (Ceuppens et al., 2011; Mazzantini et al., 2016). This involves the environment-sensing regulator proteins Fnr, ResDE, CcpA, and CodY (Duport et al., 2006; Zigha et al., 2007; van der Voort et al., 2008; Esbelin et al., 2009, 2012; Messaoudi et al., 2010) as well as other, so far less characterized two component regulatory systems (Ceuppens et al., 2011).

Consistent with international standards, routine diagnostics detect and quantify *B. cereus sensu lato* colonies on selective culture media (Ehling-Schulz and Messelhäusser, 2013). With these methods, only *presumptive B. cereus* can be detected [ISO (International Organization for Standardization) 7932:2005-03], as the members of the *B. cereus* group cannot be differentiated and the potential health risk related to a new isolate cannot be assessed. Thus, *B. cereus* isolates are additionally grown in full media such as casein glucose yeast (CGY) or brain heart infusion (BHI) supplemented with 1% glucose to determine their enterotoxin producing ability as well as cytotoxic activity by immunochemical and cell culture methods (Ehling-Schulz et al., 2011; Jeßberger et al., 2015). The data generated under these established laboratory procedures, however, do not necessarily reflect the natural conditions leading to food poisoning in humans.

Different attempts have therefore been made to investigate the behavior of *B. cereus* under simulated gastrointestinal conditions. For instance, aiming to mimic the gastric passage, media were produced consisting of gastric electrolyte solution and J broth (JB) combined with different foods (Clavel et al., 2004, 2007). Gastric and intestinal fluids were simulated by including salts, bovine serum albumin, mucin, bile salts, and digestion enzymes such as lipases, pepsin, and pancreatin (Wijnands et al., 2006, 2009). Batch culture systems were used to study anaerobiosis, low oxido-reduction potential, and carbohydrate limitation, which the bacteria are confronted with in the small intestine (Clair et al., 2010). Other *in vitro* batch cultures were developed to simulate the gastrointestinal passage in different phases, from mouth to ileum, while even competing intestinal microbiota was considered (Ceuppens et al., 2012a,b). However, hitherto only few studies focused on the interaction of *B. cereus* with human gastrointestinal cells, such as adhesion, invasion, or germination (Wijnands et al., 2007; Minnaard et al., 2013).

In the present study growth, enterotoxin production, protein secretion and cytotoxicity of a set of 19 enteropathogenic and non-pathogenic *B. cereus* strains were investigated under intestinal conditions, simulated by RPMI 1640 medium pre-incubated with the human colon epithelial cell line CaCo-2, at 37°C and 7% CO_2_. Key questions addressed are (I) the effects of the host intestine on growth, toxin gene transcription, protein secretion and enterotoxin production of the *B. cereus* strain set, (II) re-evaluation of the classification of strains as high or low toxic according to intestinal growth conditions, and (III) the distinctive characteristics of enteropathogenic and non-pathogenic strains in the intestine.

## Materials and methods

### Bacterial strains, growth conditions, and sample preparation

A set of 19 *B. cereus* strains was used for the comparative analyses carried out in frame of this study (Table 1; Jeßberger et al., 2015). Only enteropathogenic *B. cereus sensu stricto* were considered, i.e., strains negative for the emetic gene cluster *ces* and affiliated with clade I or II (Table 1; Didelot et al., 2009). The strains were assigned to different enterotoxin profiles depending on the presence of the toxin genes *hbl, nhe*, and *cytK2*. Within each toxin profile strains of different enterotoxigenic potential were chosen, including highly pathogenic (SDA KA 96, INRA C3, F837/76, NVH 0075-95, and others) and low or non-pathogenic (RIVM BC 934, F528/94, MHI 86, MHI 226, RIVM BC 90) strains (Table 1).

**Table 1:** **Nineteen ***B. cereus*** strains used in this study**.

***B. cereus* strain**	**Origin**	**Genotype clade (group)**	**Toxin gene profiling**					**CGY NheB titer**	**CGY NheB class**.	**CGY Vero tox**.	**CGY tox. class**.	**cRPMI NheB titer**	**cRPMI NheB class**.	**cRPMI CaCo-2 tox**.	**cRPMI tox. class**.
			***ces***	***hbl***	***nhe***	***cytK2***	**profile**								
14294-3 (M6)	Icecream	I (III)	−	+	+	+	A	2062	m	332	m	3074	hi	109	hi
SDA KA96	Raw milk	I (III)	−	+	+	+	A	6481	hi	1228	hi	2375	hi	158	hi
INRA A3	Starch	II (IV)	−	+	+	+	A	1299	lo	256	lo	1859	m	66	m
INRA C3	Past. carrot	II (IV)	−	+	+	+	A	4460	hi	754	hi	3276	hi	340	hi
6/27/S	Human feces	II (IV)	−	+	+	+	A	1964	m	475	m	377	lo	110	hi
F3175/03 (D7)	Human feces	II (IV)	−	+	+	+	A	5157	hi	430	m	1312	m	87	m
RIVM BC 934	Lettuce	II (IV)	−	+	+	+	A	769	lo	118	lo	713	lo	75	m
F528/94	Beef and chow mein and rice, outbreak	I (II)	−	+	+	−	C	1759	lo	214	lo	776	lo	65	m
F837/76	Human, postoperative infection	I (III)	−	+	+	−	C	8598	hi	2106	hi	2449	hi	102	hi
RIVM BC 126	Human feces	I (II)	−	+	+	−	C	7757	hi	578	hi	1433	m	116	hi
MHI86	Infant food	I (III)	−	−	+	+	D	87	lo	0	lo	19	lo	19	lo
F4429/71	Vanilla pudding	I (III)	−	−	+	+	D	4907	hi	918	hi	2146	hi	73	m
RIVM BC 964	Kebab	II (IV)	−	−	+	+	D	10266	hi	858	hi	1837	m	145	hi
F3162/03 (D8)^*^	Human feces	I (III)	−	−	+	+	D	41	lo	858	hi	1246	m	69	m
MHI226^**^	Milk and milk products	I (III)	−	−	+	−	F	930	lo	91	lo	265	lo	37	lo
NVH 0075-95	Stew with vegetables, foodpoisoning	I (III)	−	−	+	−	F	7729	hi	674	hi	2751	hi	94	m
WSBC10035	Past. milk	I (III)	−	−	+	−	F	6205	hi	1100	hi	1357	m	86	m
RIVM BC 90	Human feces	I (III)	−	−	+	−	F	146	lo	10	lo	81	lo	6	lo
7/27/S	Human feces	I (III)	−	−	+	−	F	9011	hi	952	hi	1650	m	42	m

* *Strain showed high toxicity but particularly low NheB titers in CGY due to binding failure of mAb 2B11 in sandwich EIA (Didier et al., 2015)*.

** *Sequence analysis revealed a truncated hbl operon; as strain is not able to produce Hbl L2 and Hbl B protein (negative in EIAs), it was allocated to profile F (Jeßberger et al., 2015)*.

*Genotyping of the strains had been conducted by sequence analyses of the genetic markers spoIIIAB and panC. PCR analyses with specific primers for ces and enterotoxin genes had been used for toxin profiling. A first classification of the strains as high or low toxic had been determined by sandwich EIAs against NheB and by WST-1-bioassays on Vero cells after growth of the strains under laboratory conditions (CGY medium). These preliminary data are part of and described in an earlier study (Jeßberger et al., 2015). Former classification (CGY) according to cytotoxicity was hi: >500; m: 250–500; lo: <250 and according to NheB titers hi: >4,000; m: 2,000–4,000; lo: <2,000. After growth under simulated intestinal conditions (cRPMI) in this study, NheB titers were again determined by sandwich EIAs and cytotoxicity titers were investigated in WST-1-bioassays on CaCo-2 cells. According to that, classification of the strains was adapted: cytotoxicity (hi: >100; m: 50–100; lo: <50) and NheB titers (hi: >2,000; m: 1,000–2,000; lo: <1,000)*.

All strains were pre-cultured for 17 h in casein glucose yeast (CGY) medium with 1% glucose at 37°C and 125 rpm. For simulating intestinal growth conditions, RPMI 1640 medium (with stable glutamine; Biochrom GmbH, Berlin, Germany) was used, 2% casein hydrolysate was added and total glucose content was set to 1%. Fetal calf serum (FCS) was not added due to subsequent protein analyses. Differentiated CaCo-2 cells were washed twice with PBS (PBS Dulbecco, w/o Ca^2+^, w/o Mg^2+^, low endotoxin; Biochrom GmbH, Berlin, Germany) and incubated with this RPMI 1640 medium for 22 h. Thereafter, this “CaCo-2 treated,” designated cRPMI, medium was subjected to sterile filtration using a 0.2 μm filter. The *B. cereus* strains were grown in 45 ml of cRPMI in 80 cm^2^ cell culture flasks at 37°C under 7% CO_2_ atmosphere in a cell culture incubator. Medium was inoculated to an OD_600_ of 0.05 and OD_600_ was recorded every 30 min. All strains were grown in triplicates. Eight milliliters (2 h) and six milliliters (4, 6, 8, and 10 h) samples were taken and centrifuged for 15 min at room temperature and 3,500 rpm. Cell pellets were immediately frozen at −80°C and used for determination of toxin gene transcription and intracellular protein content. Supernatant was filtered through a 0.2 μm filter, split for protein quantification as well as for the determination of enterotoxins and frozen at −20°C. For enterotoxin analyses, 1 mM EDTA was added to the supernatant.

### Cell lines and culture conditions

CaCo-2 cells were obtained from DSMZ (German Collection of Microorganisms and Cell Cultures, Braunschweig, Germany). Cells were cultivated as recommended by the supplier. RPMI 1640 medium (with stable glutamine) was supplemented with 10% fetal bovine serum (Biochrom GmbH, Berlin, Germany). The CaCo-2 cells were cultivated in 80 cm^2^ culture flasks in a humidified incubator at 37°C and 7% CO_2_ and splitted 1:6 every 3–4 days. For differentiation, 2.15 million CaCo-2 cells per flask were cultivated for 14 days gaining a cell layer mimicking the intestinal epithelium. Medium was changed every 3–4 days. To obtain cRPMI medium, cells were treated as described above.

### RNA isolation, cDNA synthesis, and quantitative real-time PCR (qRT-PCR)

RNA isolation and DNAse I digestion were performed according to Dommel et al. (2010). To compare transcript levels of all 19 *B. cereus* strains at different time points the relative quantification method according to Livak and Schmittgen (2001) was chosen. cDNA synthesis and qRT-PCR (Real-Time Quantitative Reverse Transcription PCR) were carried out as described before (Jeßberger et al., 2015). In brief, random primers (qScript cDNA Supermix, Quanta Biosciences) were used for first strand synthesis of 1 mg of total RNA. Relative gene expression was determined by qPCR and calculation via the 2^−ΔΔ*C*^T method (Livak and Schmittgen, 2001; Pfaffl et al., 2002; Lücking et al., 2009; Dommel et al., 2010). The primers used are published (Jeßberger et al., 2015). Transcription levels of the widely used house-keeping gene *rrn* (16S rRNA) served as reference for normalization applying the 2^−ΔΔ*C*^T method (Livak and Schmittgen, 2001). This method is based on the following formula: amount of target transcript = 2^−ΔΔ*C*^T with −ΔΔ*C*_T_ = − (Δ*C*_T(sample)_ − Δ*C*_T(calibrator)_) = − ((*C*_T(referencegene)_ − *C*_T(targetgene)_)_sample_ − (*C*_T(referencegene)_ − *C*_T(targetgene)_)_calibrator_). *C*_T_ showes the cycle number of the amplification reaction that exceeds the quantification threshold. Relative transcription of a target gene in % was obtained by setting the *rrn*-normalized transcription level relative to the transcript level of an external calibrator and by multiplying with 100. Target gene transcript levels of all samples tested in this study were compared with the 2^−ΔΔ*C*^T method to the expression level of *hblD* of strain F837/76 at 6 h growth in CGY medium (Jeßberger et al., 2015), which served as external calibrator and was set to 100% (log-2 = 0).

### Enzyme immunoassays (EIAs)

All monoclonal antibodies (mAbs) used for detection of *B. cereus* enterotoxin components have been generated earlier at the Department of Veterinary Sciences, Faculty of Veterinary Medicine, Ludwig-Maximilians-Universität München by immunization of mice. Production and functionality of the specific mAbs against Hbl (Dietrich et al., 1999) and Nhe (Dietrich et al., 2005) have been described in detail.

Sandwich EIAs were carried out for detection of Hbl L2 and NheB. For that, microtiter plates were coated with 100 μl/well mAb 1A12 (Hbl L2; 10 μg/ml) in bicarbonate buffer or mAb 2B11 (NheB; 5 μg/ml) in PBS, respectively. After overnight incubation at room temperature, 30 min blocking with 150 μl/well 3% sodium-caseinate-PBS and 3x washing (wash buffer: 146 mM NaCl, 0.025% Tween 20), *B. cereus* culture supernatants were applied to the microtiter plates as serial dilutions in PBS with 0.5% Tween 20. The plates were incubated for 1 h at room temperature on a tumble shaker. To avoid cross-contaminations, samples were subsequently siphoned. After four washing steps the detection antibodies were applied (100 μl/well, anti Hbl L2 8B12-HRP 1:2,000 and anti NheB 1E11-HRP 1:4,000 in 1% sodium-caseinate-PBS). After 1 additional h at room temperature on a tumble shaker and 5 further washing steps, 100 μl/well 5% TMB (tetramethylbenzidin)-solution in citrate buffer were applied. The reaction was stopped after 20 min by addition of 1 M sulfuric acid and absorbance at 450 nm was measured immediately in a Tecan photometer using Ridawin software. Titers are defined as the reciprocal of the highest dilutions resulting in an absorbance value of ≥1.0.

Indirect EIAs were performed for detection of enterotoxin components Hbl L1 and Hbl B. The microtiter plates were coated with serial dilutions of the *B. cereus* culture supernatants in bicarbonate buffer. The mAbs 1E9 (Hbl L1; 1 μg/ml in PBS) and 1B8 (Hbl B; 2 μg/ml in PBS) served as primary antibodies. For detection, a polyclonal rabbit-anti-mouse-HRP conjugate was applied (1:2,000 in 1% sodium-caseinate-PBS). Washing and incubation steps were carried out analogously to the sandwich EIAs.

As an exception, for detection of NheB of strain F3162/04 (D8) the indirect assay solely based on mAb 1E11 was applied (Jeßberger et al., 2015).

### Quantification of total protein amount

The extracellular protein contents were determined from *B. cereus* culture supernatants. Protein amounts were quantified by Roti-Nanoquant Kit (Roth, Karlsruhe, Germany) in microtiter plates according to the manufacturer's instructions. Colorimetric reactions were measured with Infinite F200 reader (Tecan) at wavelengths of 610/450 nm. Protein concentrations were determined by quotient of optical densities OD 610/450 referred to an internal standard generated by Quick Start Bovine Serum Albumin Standard (Biorad).

### Cytotoxicity assays

WST-1 bioassays on CaCo-2 cells were performed as previously described (Didier et al., 2012; Jeßberger et al., 2014, 2015). Briefly, serial dilutions of the *B. cereus* culture supernatants in RPMI 1640 medium were applied to 96 well plates (100 μl/well). One hundred microliters/well CaCo-2 cell suspensions (2 × 10^4^ cells/well) were added immediately. After 24 h incubation at 37°C and 7% CO_2_, cell viability was determined by addition of WST-1 (Roche diagnostics). Optical density was recorded in a Tecan photometer at 450 nm. Dose-response curves and thus 50% lethal concentrations were calculated with Ridawin software and are shown as reciprocal titers.

Propidium iodide influx tests (Jeßberger et al., 2014, 2015) were used to assess pore formation in the membranes of CaCo-2 cells. For that, 4 × 10^4^ CaCo-2 cells were seeded in 200 μl RPMI 1640 medium/well in 96 well plates and incubated for 24 h at 37°C and 7% CO_2_. After that, 100 μl medium were removed and 100 μl fresh RPMI 1640 medium were added containing 10 μg/ml PI (Sigma-Aldrich) and 1:20 dilutions of the *B. cereus* culture supernatants. Subsequently, fluorescence was measured in a Victor 1420 multilabel counter (Perkin Elmer) for 4 h every 2.5 min (excitation: 530 nm; emission: 616 nm; excitation time: 1 s; excitation strength: 20,000). Fluorescence curves were calculated using Microsoft Excel and samples were compared according to the highest linear slope of these curves.

## Results

### Similar growth behavior under simulated intestinal conditions

**Figure 1:**
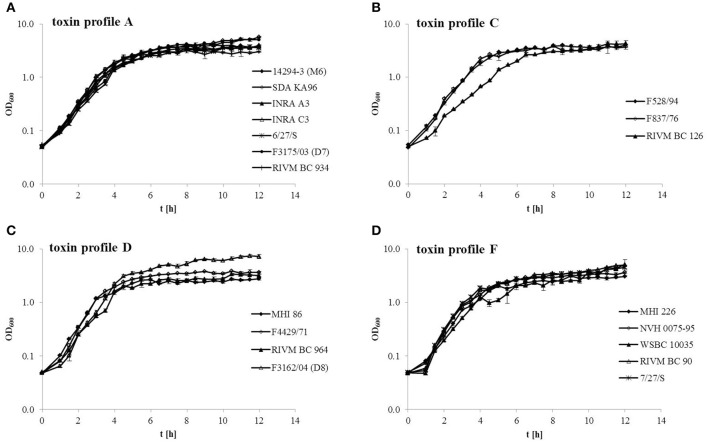
**Growth of 19 ***B. cereus*** strains under simulated intestinal conditions (RPMI medium treated with CaCo-2 cells, 37°C, 7% CO_**2**_)**. Strains are grouped consistent with their toxin profile. **(A)** Toxin profile A (*nhe, hbl, cytK2*). **(B)** Toxin profile C (*nhe, hbl*). **(C)** Toxin profile D (*nhe, cytK2*). **(D)** Toxin profile F (*nhe*).

The 19 *B. cereus* strains were compared regarding their growth behavior under simulated intestinal conditions (in CaCo-2 treated cRPMI medium at 37°C and 7% CO_2_) (Figure 1). All strains of toxin profile A grew quite similar. Only SDA KA96 reached a higher OD_600_ (>5) than the remaining strains (OD_600_ 3–4) (Figure 1A). In toxin profile C RIVM BC 126 showed delayed growth but a similar OD_600_ (3.7–4.3) than the remaining strains (Figure 1B). This strain was isolated from human feces, as was F3162/04 (D8), which grew to significantly higher OD_600_ (>7 compared to 2.7–3.6) than all other strains in toxin profile D (Figure 1C). For toxin profile F, similar growth of all strains was observed. NVH 0075-95 and the non-pathogenic strain MHI 226 showed slightly reduced OD_600_ (>3 compared to 4.5–5) than the remaining strains (Figure 1D).

### Early toxin gene transcription under simulated intestinal conditions

qRT-PCR was used to determine transcription of the enterotoxin genes *nheB* (NheB) and *hblD* (Hbl L1) of all 19 *B. cereus* strains grown under simulated intestinal conditions. Transcription was analyzed after 2, 4, 6, 8, and 10 h of growth.

**Figure 2:**
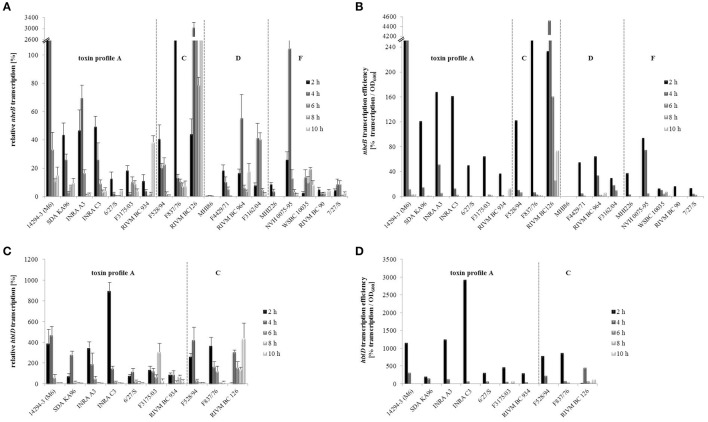
**Enterotoxin gene transcription of the ***B. cereus*** strains**. *nheB* **(A)** and *hblD* **(C)** transcription was determined by qRT-PCR, normalized to 16S *rrn* levels of the same sample and relative to the transcript level of an external calibrator. *hblD* expression of reference strain F837/76 at 6 h growth in CGY medium (Jeßberger et al., 2015) was used as calibrator and set to 100% (log-2 = 0). With the 2^−ΔΔ*C*^T method, transcription of all other samples was compared to this calibrator. Transcription efficiencies of *nheB* **(B)** and *hblD* **(D)** were determined as relative transcript level/OD_600_. Strains are grouped according to their enterotoxin gene profiles, which are separated by dotted lines.

Relative *nheB* transcription was particularly high in strains 14294-3 (M6) and RIVM BC 126 compared to all other strains (Figure 2A). With the exception of RIVM BC 126, *nheB* transcription efficiency (relative transcript level/OD_600_) of all strains was highest after 2 h growth. At later time points, transcription efficiency decreased significantly (Figure 2B). For 8 out of 10 strains, relative *hblD* transcription was higher than relative *nheB* transcription (Figure 2C). Strain INRA C3 showed the maximum relative *hblD* transcription at 2 h growth. For 9 out of 10 strains, *hblD* transcription efficiency was also highest after 2 h growth (Figure 2D).

It has been shown before that the toxic activity of a *B. cereus* isolate grown in CGY medium correlates with its ability to produce NheB and Hbl L1/B protein (Moravek et al., 2006; Jeßberger et al., 2014). Generally, enterotoxin gene transcription under simulated intestinal conditions only partially correlated with the former classification of the strains as high or low NheB producing and high or low toxic (Figure 2 and Table 1). Setting a random threshold of 20% relative *nheB* transcription, the following strains can be considered to transcribe high levels of *nheB*: the formerly classified as high toxic strains SDA KA 96, INRA C3, F837/76, RIVM BC 126, RIVM BC 964, F3162/04 (D8), and NVH 0075-95, but also the formerly low or medium toxic strains 14294-3 (M6), INRA A3, and F528/94. The formerly classified as low or non-pathogenic strains MHI 86, MHI 226, and RIVM BC 90 transcribed comparatively low levels of *nheB* under simulated intestinal conditions.

When a random threshold of 150% for *hblD* transcription was set, 8 out of 10 strains can be considered to transcribe high levels of *hblD*, among them 5 formerly high and 3 formerly low toxic strains.

### Strain-specific enterotoxin production starts early under simulated intestinal conditions

**Figure 3:**
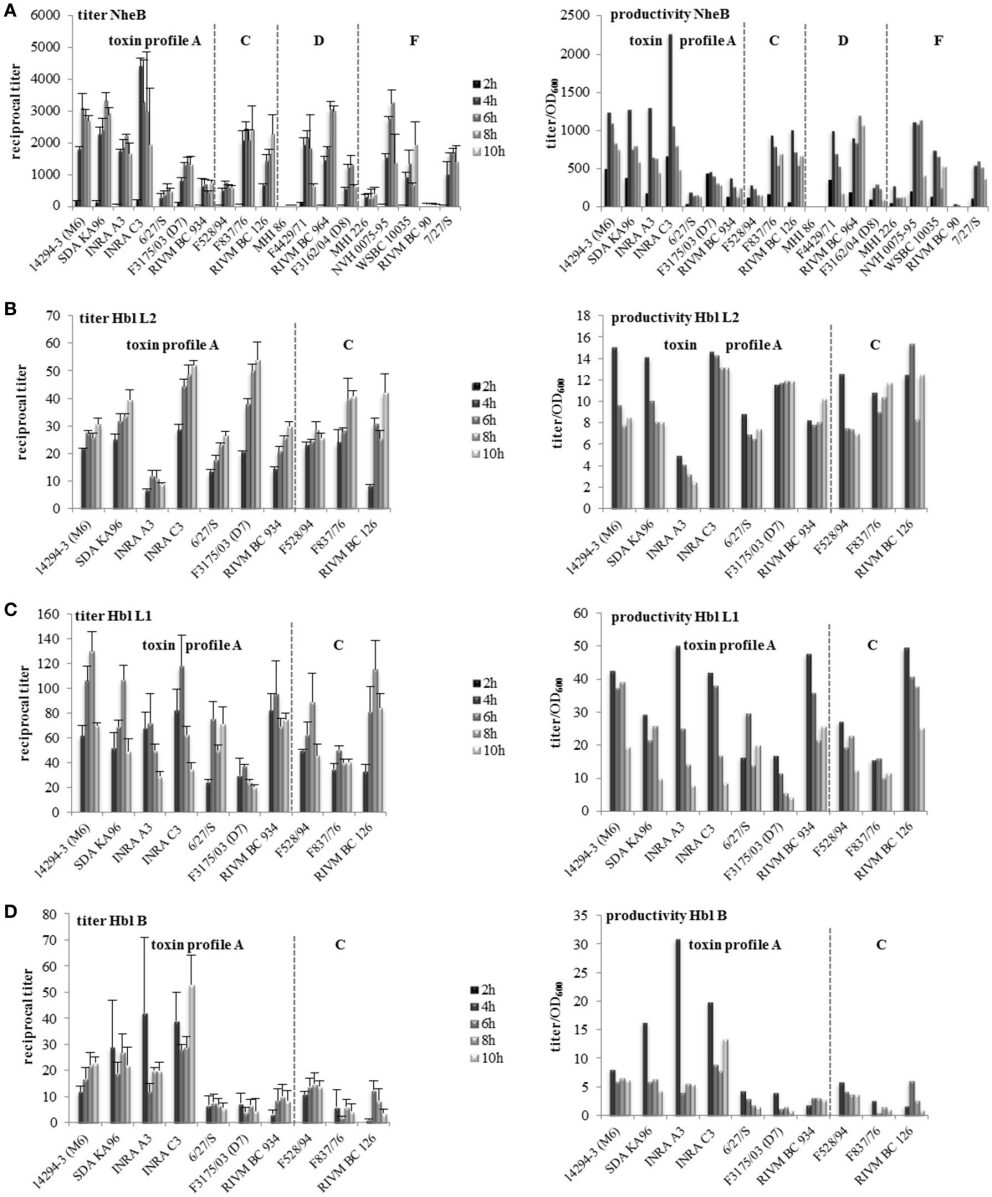
**Enterotoxin production of 19 ***B. cereus*** strains grown under simulated intestinal conditions**. Reciprocal titers as well as the productivity (titer/OD_600_) are shown. **(A)** NheB. **(B)** Hbl L2. **(C)** Hbl L1. **(D)** Hbl B. Strains are grouped according to their enterotoxin gene profiles, which are separated by dotted lines.

Growth of the *B. cereus* strains under simulated intestinal conditions resulted in strain specific enterotoxin production, measured with specific EIAs (Dietrich et al., 1999, 2005; Jeßberger et al., 2015). Enterotoxin titers as well as productivity (titer/OD_600_) are shown in Figure 3. Generally all titers were significantly lowered compared to growth of the *B. cereus* strains in CGY medium (Jeßberger et al., 2015), due to reduced growth under the defined intestinal conditions. Interestingly, NheB was detectable already after 2 h of growth (Figure 3A). After 4 h, comparably high NheB titers were measured; for 8 out of 19 strains these titers did not increase after prolonged growth. The formerly classified as non-pathogenic strains MHI 86 and RIVM BC 90 showed the lowest NheB titers, followed by MHI 226. Calculating enterotoxin productivity (titer/OD_600_) demonstrated that NheB production/cell was most efficient at 4 h. On the other hand, with the exception of F4429/71, titers did also not significantly decrease over the time, indicating that only little proteolytic degradation takes place under simulated intestinal conditions.

The Hbl components L2, L1, and B were not detectable after 2 h. Except RIVM BC 126, all *hbl* positive strains produced comparably high amounts of Hbl L2 after 4 h, which even increased over time (Figure 3B). The highest titers were measured after incubation for 10 h. Nevertheless, Hbl L2 productivity, i.e., titers determined per OD_600_, was generally highest after 4 h. Hbl L1 titers of 6 out of 10 strains increased significantly over time, but productivity was again highest at 4 h (Figure 3C). Five out of ten strains showed significantly reduced titers after 10 h, suggesting that the latter enterotoxin component might be more susceptible to proteolytic degradation. For Hbl B comparably high titers were detected after 4 h, which weren't significantly increased over time. Only strain RIVM BC 126 showed a significant increase of the Hbl B titer from 4 to 6 h.

### Protein secretion starts early under simulated intestinal conditions

**Figure 4:**
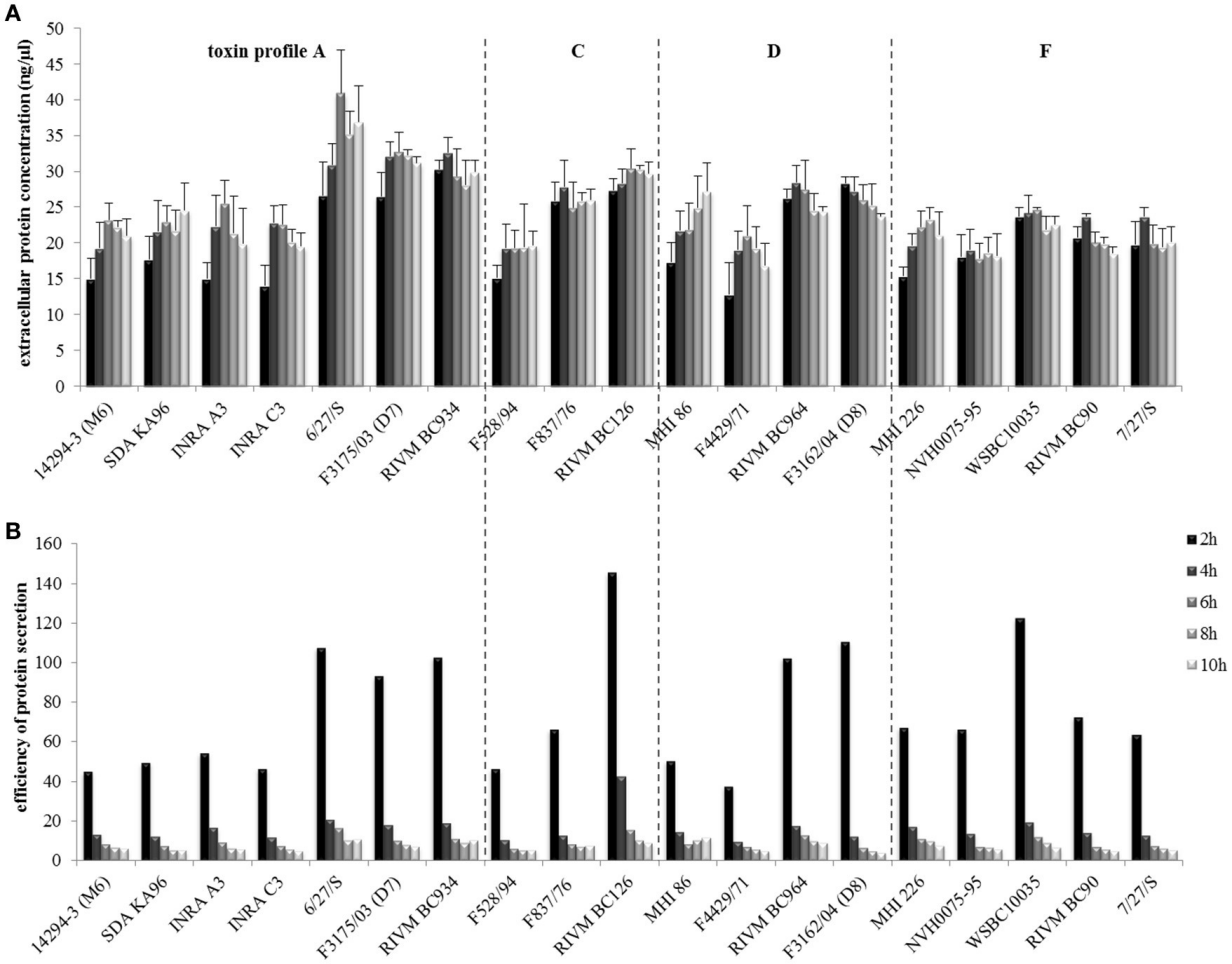
**Total protein secretion of the ***B. cereus*** strains grown under simulated intestinal conditions. (A)** Quantification of extracellular protein after 2, 4, 6, 8, and 10 h growth. **(B)** Efficiency of protein secretion determined by normalization of extracellular protein concentrations to the OD_600_. Strains are grouped according to their enterotoxin gene profiles, which are separated by dotted lines.

Total protein concentrations of all strains were determined as described previously (Jeßberger et al., 2015). After only 2 h growth, extracellular proteins were already detectable in all strains at average concentrations of 20–25 ng/μl (Figure 4A). The highest extracellular protein concentrations were found in the supernatant of strain 6/27/S. To assess the efficiency of protein secretion, extracellular protein concentration was normalized to the optical density (OD_600_). All strains showed highest secretion efficiency after 2 h growth, with RIVM BC 126 being the most efficiently protein secreting strain (Figure 4B). Intracellular protein concentrations were comparable among all tested strains (data not shown).

### Despite reduced growth, toxin gene transcription, enterotoxin production, and protein secretion are enhanced under simulated intestinal conditions compared to “standard” laboratory cultivation

Due to oxygen and nutrient limitation, growth of all 19 strains was decreased under simulated intestinal conditions compared to laboratory conditions (Figure 1 and Jeßberger et al., 2015). While in CGY medium strains grew to maximum optical densities of 20 (Jeßberger et al., 2015), the average maximum OD_600_ in cRPMI was 4–5. Log phases were shortened (2–4 h in cRPMI compared to 2–6 h in CGY). While strain MHI 226 showed decreased growth in CGY, this was no longer observed in cRPMI. On the other hand, in cRPMI, strain RIVM BC 126 grew significantly slower than the other strains of toxin profile C, while strain F3162/04 (D8) grew to a final OD_600_ significantly higher than all other strains.

**Figure 5:**
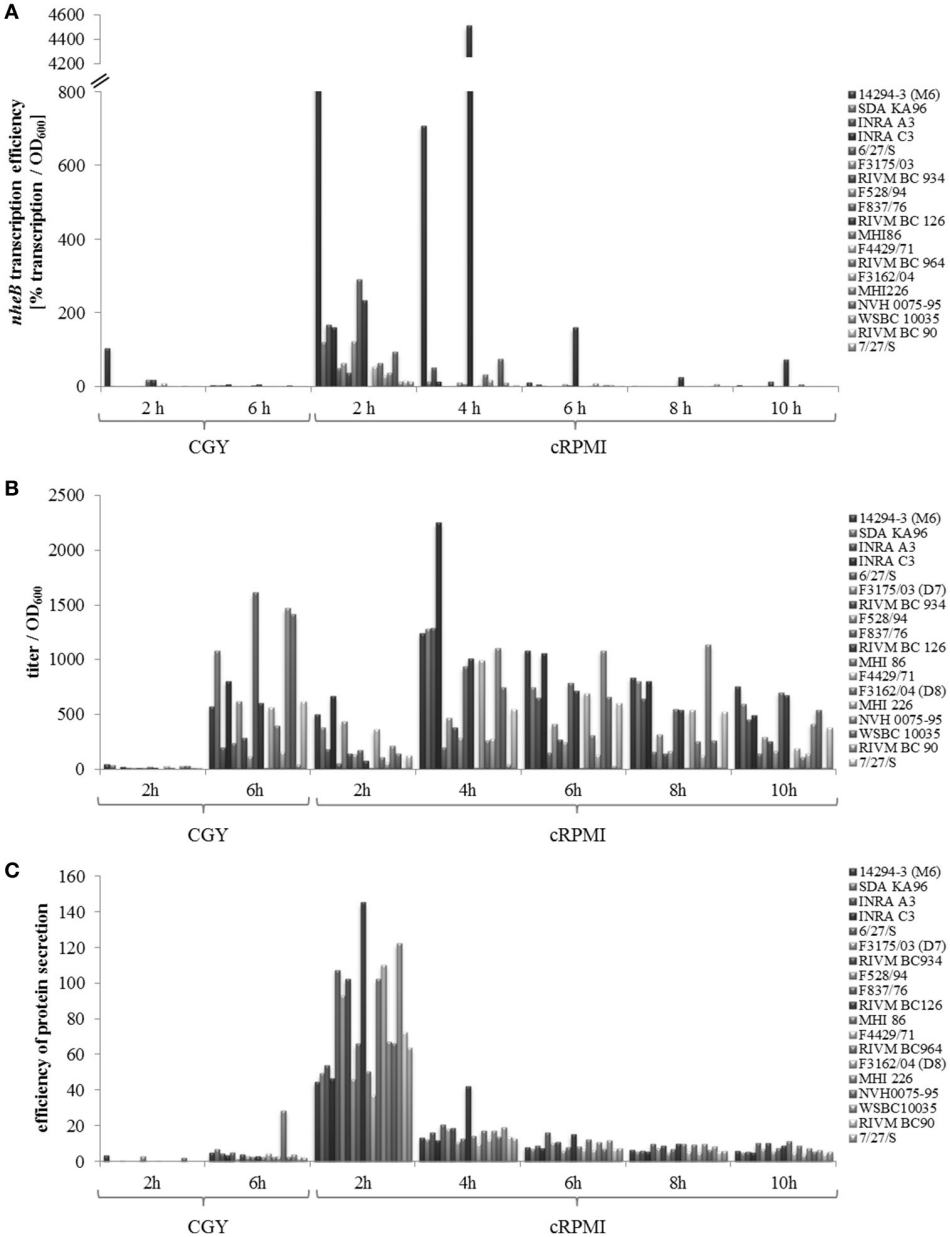
**Comparison of the ***B. cereus*** strain set grown in CGY (Jeßberger et al., **2015**) and cRPMI (this study). (A)** Transcription efficiency of *nheB* determined as % transcription per OD_600_. **(B)** NheB productivity determined as reciprocal titer per OD_600_. **(C)** Efficiency of protein secretion determined as extracellular protein concentration per OD_600_.

Figure 5 compares efficiency of toxin gene transcription (*nheB*), enterotoxin productivity (NheB), and protein secretion efficiency of the 19 *B. cereus* strains grown in CGY medium (Jeßberger et al., 2015) and under simulated intestinal conditions (this study). Enterotoxin gene transcription turned out to be strain specifically activated after incubation for 2 h under simulated intestinal conditions (Figure 5A). Up to 40-fold increased amount of NheB toxin was found in the supernatant of all strains grown for 2 h in cRPMI compared to CGY (Figure 5B). This might partially result from enhanced *nheB* transcription, but also from generally increased protein secretion (Figure 5C). Likewise to standard laboratory conditions (Jeßberger et al., 2015), our current work showed that even under simulated intestinal conditions enterotoxin gene transcription provides no reliable information about the toxic potential of a *B. cereus* isolate, which points toward additional posttranscriptional and posttranslational regulatory mechanisms.

### Enhanced enterotoxin production results from pre-incubation with the CaCo-2 cells

**Figure 6:**
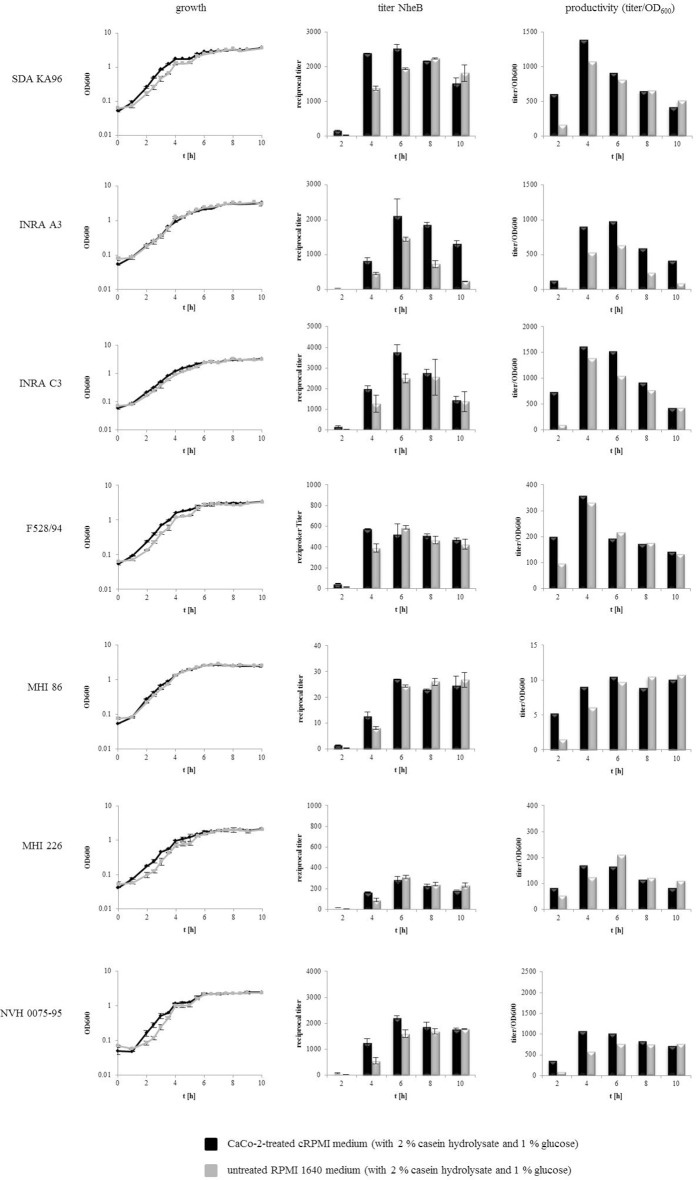
**Growth and enterotoxin production of 7 selected ***B. cereus*** strains comparing CaCo-2 treated cRPMI medium with untreated RPMI 1640 medium**. Both media types were supplemented with 2% casein hydrolysate and 1% glucose. cRPMI medium had been pre-incubated for 22 h with differentiated CaCo-2 cells. Bacteria were grown in 80 cm^2^ cell culture flasks at 37°C under 7% CO_2_ atmosphere. OD_600_ was recorded for 10 h and every 2 h samples of the supernatant were taken. Enterotoxin production was determined via sandwich EIA specific for NheB.

Prior experiments showed an increase of enterotoxin production and protein secretion under the chosen simulated intestinal growth conditions compared to standard laboratory conditions (Figure 5). Thus, the question arose whether this was due to the change of growth conditions (increased temperature, 7% CO_2_ atmosphere, no agitation, nutrient limitation) or due to the pre-incubation of the medium with CaCo-2 cells. To examine this, seven selected *B. cereus* strains were compared in CaCo-2 treated cRPMI medium and in untreated RPMI 1640 medium under otherwise identical conditions in the cell culture incubator. While three strains showed no growth differences in the two compared media, four strains had a slightly accelerated log phase in the CaCo-2 treated cRPMI medium (Figure 6). All of the tested strains showed enhanced enterotoxin production (represented by reciprocal titers of the toxin component NheB as well as by calculation of the productivity [titer/OD_600_]) in the CaCo-2 treated cRPMI medium at early time points (2, 4, and partially 6 h after inoculation). After longer incubation times, NheB titers of six out of seven strains grown in the untreated RPMI 1640 medium caught up (Figure 6). This experiment shows that under both conditions enterotoxin production takes place, but that it is enhanced in the CaCo-2 treated cRPMI medium at early time points. Thus, it can be concluded that the signal triggering early enterotoxin production is present in the medium and indeed originates from the CaCo-2 cells.

### Strain specific cytotoxicity

**Figure 7:**
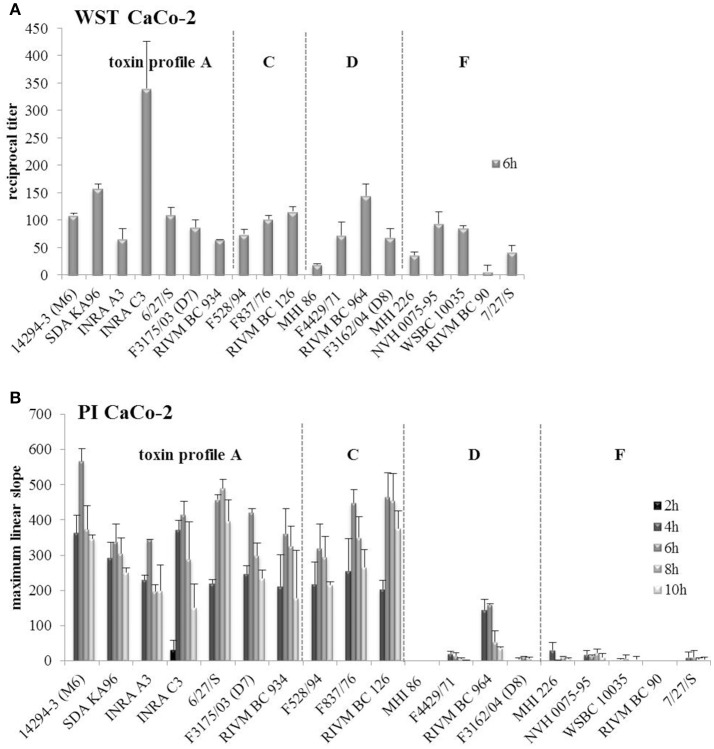
**Toxicity tests of the 19 ***B. cereus*** strains on CaCo-2 cells. (A)** Reciprocal titers for 50% lethal doses obtained in WST-1 bioassays are shown. The assay was performed only after 6 h of growth under simulated intestinal conditions. **(B)** Propidium iodide influx tests were performed after 2, 4, 6, 8, and 10 h of growth. The maximum linear slope of the fluorescence curves of each strain is shown. Strains are grouped according to their enterotoxin gene profiles, which are separated by dotted lines.

For cytotoxicity assays, the human colon carcinoma cell line CaCo-2 was used. Due to reduced growth under simulated intestinal conditions and thus, reduced amounts of enterotoxins (Figures 1, 3), reciprocal titers obtained in WST-1 bioassays (Figure 7A) decreased compared to earlier tests (Jeßberger et al., 2015), and differences between strains formerly classified as high and low toxic seemed to be less distinct. Also pace of pore formation by the enterotoxins, determined by propidium iodide influx tests, was slightly decreased compared to earlier experiments (see Figure 7B and Jeßberger et al., 2015). Nevertheless, it became obvious that *hbl* positive strains caused much more rapid pore formation than solely Nhe producing strains, a fact that has been observed before (Jeßberger et al., 2014, 2015), even when growth and as a result enterotoxin (Hbl) production was limited. After 2 h no toxic effect was seen, and for 5 out of 10 *hbl* positive strains pace of PI influx was increased from 4 h to later time points.

## Discussion

To determine the toxic potential of a *B. cereus* isolate, bacteria are usually cultivated under laboratory conditions, which stimulate maximal growth and thus enhance the amount of secreted toxins (Jeßberger et al., 2015). These procedures, however, do not match the situation in the human gastrointestinal tract where the bacteria are enfacing nutrient limitation and anaerobic or micro-aerobic conditions (Clair et al., 2010). Hitherto, no suitable animal system is established to simulate growth behavior and enterotoxin production of *B. cereus* in the human intestine. In search of a relatively simple and easy to handle *in vitro* system simulating the human intestine, we applied “CaCo-2 treated” cRPMI medium, which was pre-incubated with the human epithelial colorectal adenocarcinoma cell line CaCo-2.

Routinely, the cytotoxic potential of a new *B. cereus* isolate is determined in WST-1 bioassays on Vero cells (Dietrich et al., 1999; Moravek et al., 2006; Jeßberger et al., 2015). Titers are determined as reciprocal value of the supernatant dilution that results in 50% loss of mitochondrial activity. High titers represent high toxic potential. The comparison of a variety of *B. cereus* isolates enables their classification as high, medium or low toxic (see Table 1). Vero, an epithelial cell line from the kidney of the African green monkey, is often used because of its high susceptibility toward bacterial toxins (Miyamura et al., 1974; Yutsudo et al., 1987). A recent study showed that *B. cereus* enterotoxins are harmful to a variety of different cell lines. Of these, CaCo-2 cells seem to be best suited to simulate the human intestine, although they are generally less susceptible and respond rather to Hbl than to Nhe in comparison to Vero cells (Jeßberger et al., 2014). When the *B. cereus* isolates were cultivated under simulated intestinal conditions and CaCo-2 were used as target cells, the criteria for the classification as high, medium or low toxic (see Table 1 and Jeßberger et al., 2015) had to be adjusted, as otherwise most of the strains were underestimated. Thus, in cRPMI, more strains were now classified as medium toxic (Table 1), which should still be considered potentially dangerous. The general tendency of high, medium, or low toxicity remained, whether the strains were cultivated in CGY or cRPMI (Table 1), but under simulated intestinal conditions the differences between high and low toxic strains were decreased (Figure 7).

This study has clearly shown that enterotoxin gene expression, enterotoxin production, and total protein secretion are enhanced and start extremely early under the chosen simulated intestinal conditions. This might be due to time limitation, as the bacilli pass the human intestine to a large extent (Camilleri et al., 1989).

Our data further revealed a discrepancy between enterotoxin gene transcription and enterotoxin titers in the culture supernatants (compare Figures 2, 3). This had also been observed when the analyses were performed after growth of the strains under laboratory conditions (CGY medium). At that time it was concluded that further posttranscriptional and posttranslational processes might be involved, such as alternative regulation by non-coding RNA (riboswitches) (Jeßberger et al., 2015). Unusually long 5′ untranslated regions (UTR) upstream of the start codons of both the *nhe* and the *hbl* operon have been identified (Böhm et al., 2016) suggesting posttranscriptional regulation via formation of regulatory mRNA structures. An involvement of riboswitches in gene expression has already been demonstrated for *B. subtilis* and *B. anthracis* (Welz and Breaker, 2007; Wilson-Mitchell et al., 2012). Furthermore, posttranslational regulation may also add to the complex regulatory network of enterotoxin gene expression, as it was recently shown for the regulation of cereulide toxin production in emetic *B. cereus* (Ehling-Schulz et al., 2015; Lücking et al., 2015; Kranzler et al., 2016). Overall, these multiple levels of regulation complicate the predictability of enterotoxin production.

Stimulation by intestinal conditions has also been observed for *Clostridium perfringens*, another Gram positive, toxin producing species. Compared to *in vitro* growth, rapid upregulation of toxin genes in the presence of CaCo-2 cells was observed, followed by enhanced protein secretion. This was detectable after only 1 h of infection and even enhanced after 2 and 3 h (Vidal et al., 2009). When growth and toxin production of selected *B. cereus* strains were compared in CaCo-2 treated cRPMI medium and in untreated RPMI 1640 medium under otherwise identical conditions, enhanced and early enterotoxin production was observed in the presence of the CaCo-2 cells or their supernatant (Figure 6). Thus, we concluded that a soluble factor present in the medium and originated from the CaCo-2 cells stimulates toxin production. Considering that enterotoxin titers of six out of seven strains in untreated RPMI 1640 medium converged with those in cRPMI after 6–8 h (Figure 6), one could also speculate that the factor accelerating toxin production is used up by the bacteria. On the contrary, only pre-infected CaCo-2 cells triggered toxin production of *C. perfringens* (Vidal et al., 2009). It was concluded that rapid host-cell stimulated secretion of most *C. perfringens* toxins is triggered by an unknown host factor present during infection and that close contact between the bacteria and the host cells is required, as the factor is Caco-2-surface-bound (Vidal et al., 2009). In both cases, the signal (factor) stimulating the bacteria could not be further localized. It has been suggested that a lack of glucose activates *B. cereus* enterotoxin gene transcription *via* CcpA-dependent catabolite repression (van der Voort et al., 2008). Hence, we measured glucose concentrations in cRPMI for 36 h. It appeared that glucose was not used up until the bacilli reached the end of the exponential growth phase (data not shown). So it is assumed that enterotoxin production of *B. cereus* is stimulated by a so far unidentified host factor. In an earlier study it has been observed that germination of 8 out of 11 *B. cereus* strains was induced by CaCo-2 cells and also by their (heated) supernatant. Thus, it was concluded that this heat stable germinant is released by the eukaryotic cells and thereupon bound or degraded by the *B. cereus* spores (Wijnands et al., 2007).

Activation of virulence factor gene expression and of protein secretion upon host contact has been primarily described for invasive pathogens. It has been reported that contact with epithelial cells induces transient assembly of appendages on the surface of *Salmonella typhimurium* (Ginocchio et al., 1994). After host cell contact and initial formation of A/E (attaching and effacing) lesions, enteropathogenic *Escherichia coli* (EPEC) increase transcription of genes involved in adherence and virulence (Leverton and Kaper, 2005). For example EspC (autotransporter protein) secretion is stimulated when bacteria are grown in cell culture medium and increased in the presence of epithelial cells (Vidal and Navarro-García, 2006). Transcription of virulence genes of *Shigella flexneri* has been found to be transiently regulated by the type III secretion machinery upon entry into epithelial cells (Demers et al., 1998). After contact with host cells *Helicobacter pylori* produces parts of the Hp T4SS (*Helicobacter pylori* type IV secretion system) and activates two different invasion mechanisms (Rohde et al., 2003). Our study showed that contact with the CaCo-2 treated medium enhances enterotoxin gene transcription as well as total protein secretion of *B. cereus*. Up to now it is not quite clear how the main virulence factors, the enterotoxin complexes Nhe and Hbl, are secreted. The occurrence of Sec-type secretion signal peptides at the proteins' N-terminus points to secretion via the *sec* pathway (Fagerlund et al., 2010; Vörös et al., 2014), but evidence has also been found for involvement of flagellar export complexes similar to type III secretion systems of Gram negative bacteria (Senesi and Ghelardi, 2010). In contrast to invasive pathogens, according to our data *B. cereus* does not necessarily need close contact to the host cells. Sensing the intestinal environment seems to be sufficient for stimulating the production of enterotoxins, which subsequently attack the host cells via ingenious mechanisms of pore formation (Heilkenbrinker et al., 2013; Didier et al., 2016; Zhu et al., 2016).

## Conclusion

As revealed by our study, cultivation of *B. cereus* under “standard” laboratory conditions does not allow conclusive predictions of the toxic potential of enteropathogenic *B. cereus* strains in the human intestine. Simulated intestinal growth conditions, such as the ones presented here, have to be included in the standard cultivation procedure. cRPMI medium, pre-incubated with host cells, was shown to accelerate and enhance enterotoxin production per cell. The cRPMI medium represents an interesting alternative for the determination of the enterotoxigentic potential of *B. cereus* strains in a more host simulating setting, as long as the signal originating from the CaCo-2 cells, which stimulates enterotoxin production, is unknown. Elucidating the latter one is subject of ongoing research.

## Author contributions

NJ performed growth experiments and sample preparation and wrote the manuscript. VK and MB carried out transcription experiments. CR carried out the protein secretion studies. AM and NJ determined enterotoxin production and cytotoxicity. RD, SS, ME, and EM were involved in experimental setup and writing of the manuscript.

### Conflict of interest statement

The authors declare that the research was conducted in the absence of any commercial or financial relationships that could be construed as a potential conflict of interest.
